# RNF183 Is a Prognostic Biomarker and Correlates With Tumor Purity, Immune Infiltrates in Uterine Corpus Endometrial Carcinoma

**DOI:** 10.3389/fgene.2020.595733

**Published:** 2020-11-26

**Authors:** Rong Geng, Yuhua Zheng, Lijie Zhao, Xiaobin Huang, Rong Qiang, Rujian Zhang, Xiaoling Guo, Ruiman Li

**Affiliations:** ^1^Department of Gynecology and Obstetrics, The First Affiliated Hospital, Jinan University, Guangzhou, China; ^2^Department of Gynecology, Affiliated Foshan Maternity & Child Healthcare Hospital, Southern Medical University, Foshan, China; ^3^Foshan Maternal and Children Healthy Research Institute, Affiliated Foshan Maternity & Child Healthcare Hospital, Southern Medical University, Foshan, China

**Keywords:** estrogen receptor alpha, immune infiltration, prognosis, uterine corpus endometrial carcinoma, RNF183

## Abstract

RNF183, a member of the E3 ubiquitin ligase, has been shown to involve in carcinogenesis and proposed as one of the biomarkers in Uterine Corpus Endometrial Carcinoma (UCEC). However, no research focused on the role of RNF183 in UCEC. We analyzed the expression and immune infiltration of RNF183 in UCEC. TIMER, UALCAN, and GEPIA were used to analyze the gene expression of RNF183. We emplored Kaplan-Meier Plotter to examine the overall survival and progression-free survival of RNF183, and applied GeneMANIA to identify RNF183-related functional networks. LinkedOmics was helpful to identify the differential gene expression of RNF183, and to further analyze gene ontology and the genome pathways in the Kyoto Protocol. Finally, we used TIMER to investigate the immune infiltration of RNF183 in UCEC. Otherwise, we partly verified the results of bioinformatics analysis that RNF183 controlled ERα expression in ERα-positive Ishikawa cells dependent on its RING finger domain. We also found that ERα increased the stability of RNF183 through the post-translational mechanism. Together, patients with a high level of RNF183 harbor favorable overall and progression-free survival. High expression of RNF183 was associated with a low stage, endometrioid, and TP53 Non-Mutant status in endometrial cancer. The RNF183 expression was greater at higher expression and the tumor stage was greater at the lower level. On the side of immunization, high level of RNF183 in UCEC is negatively related to tumor purity, infiltrating levels of CD4 + T cells, neutrophils, and dendritic cells. Besides, the expression of RNF183 in UCEC is significantly correlated with the expression of several immune cell markers, including B cell, M1 macrophage marker, M2 Macrophage, Dendritic cell, Th1 markers, Th2 markers, Treg markers, and T cell exhaustion markers, indicating its role in regulating tumor immunity. These results suggested that RNF183 may be considered as a novel prognostic factor in endometrial cancer and an early diagnostic indicator for patients with UCEC.

## Introduction

Uterine Corpus Endometrial Carcinoma (UCEC) is the fourth most common gynecological malignancy in developed countries (Kandoth et al., 2013), and the incidence has been raised rapidly in China, increasing 63,400 new cases a year (Chen et al., 2016). According to biological and histopathological variables, endometrial cancer is classified into two types. Type II tumors are usually poorly differentiated, non-endometrioid, and more likely to metastasis, relapse even after aggressive clinical intervention. By contrast, type I endometrial cancer is often endometrioid and well-differentiated, presumably owing to greater exposure to a long history of unopposed estrogen or other risk factors inducing hyperestrogenism such as obesity. Endometrial cancer is one of the few human malignant tumors for which mortality is increasing (Berg et al., 2017), which underlines the urgency to develop more effective methods for the early diagnosis and treatment of this disease.

The RNF183 (RING finger 183) is served as an E3 ubiquitin ligase (E3_*s*_) belonged to the RING finger protein family. RING finger domain has been characterized by the sequence of CX2CX(9–39)CX(1–3)HX(2–3)C/HX2CX(4–48) primarily responsible for substrate specific identification in ubiquitylation (Joazeiro and Weissman, 2000; Lipkowitz and Weissman, 2011). RING finger ubiquitin ligases are involved in the process of essential cellular functions, such as maintaining the integrity of genomic, cell cycle, cell signal, and DNA repair. For example, the FANC core complex containing RING finger-like PHD domain. Its mutation induces Fanconi anemia which increases the risk of cancer (Moldovan and D’Andrea, 2009). Besides, MDM2 targets tumor suppressor p53 for degradation (Oliner et al., 1992; Wade et al., 2010). Inactivated RING finger E3s BRCA1 destroys the DNA repair pathway in breast and ovarian cancer (Hashizume et al., 2001; Ruffner et al., 2001). Properly, RING finger E3s are involved both in the promotion and the suppression of cancers. Distinct RING finger E3s are particular therapeutic targets. Small molecular inhibitors suppress the MDM2–p53 interaction in preclinical studies. Accumulation functional and controlled pathway data from RING finger E3s are helpful for developing new targeted therapy.

RNF183, RNF182, RNF186, and RNF152, are further identified as the RNF183 family, which share the similar structure RING finger domain (C3HC4) at their N-terminus and transmembrane domains at their C-terminus with high homology (Kaneko et al., 2016; Okamoto et al., 2020a). As common features, members of RNF183 family have exhibited a broad range of functions in diverse biological and pathological processes such as prolonged endoplasmic reticulum stress, apoptosis, ischemia-reperfusion injury, oxygen, and glucose metabolism, immune and inflammatory response (Liu et al., 2008; Nectoux et al., 2010; Wang et al., 2018; Wu et al., 2018; Cao et al., 2019; Maeoka et al., 2019a). It was proposed that RNF183 could be as one of the potential biomarkers for endometrial cancer through gene expression screening (Colas et al., 2011). However, the RNF183 involvement of molecular mechanisms underlining the disease remains unclear.

Here we find that RNF183 is upregulated in endometrial cancer and mostly higher in endometrioid, low-grade, TP53-Non-Mutant samples. It is also negatively related to tumor purity, infiltrating levels of CD4^+^ T cells, neutrophils, and dendritic cells. Besides, the RNF183 in UCEC is significantly correlated with the expression of several immune cell markers, including B cell, M1 macrophage marker, M2 Macrophage, Dendritic cell, Th1 markers, Th2 markers, Treg markers, and T cell exhaustion markers. For mechanism, RNF183 shows a significant correlation with ERα. We prove that RNF183 regulates ERα and ERα target genes under the existence of the RING finger domain. Furthermore, ERα promotes the stability of RNF183.

## Materials and Methods

### UALCAN Database

UALCAN^1^ (Chandrashekar et al., 2017) is a cancer data online analysis, mainly based on the TCGA level 3 RNA—seq and clinical data of 31 types of cancer in 74 samples of normal and tumor by the relative expression of genes. The database can be spectrum identification of target gene expression, DNA promoter region methylation analysis, survival analysis and correlation analysis. It also can check other related information in the database through the link. For example, gene modification and miRNA prediction were examined.

### GEPIA Database

Gene Expression Profiling Interactive Analysis (GEPIA) database^2^ is used to analyze the RNA sequencing expression data of 8,587 healthy and 9,736 tumor tissue samples from TCGA and GTEx projects (Tang et al., 2017) including single-gene analysis, cancer type analysis, and polygene analysis. By inputting the target gene on this website, the differential expression, survival analysis, correlation analysis and PCA of the target gene can be obtained. We generated the expression of the RNF183 gene through GEPIA.

### Kaplan-Meier Plotter Database

Kaplan-Meier survival curve analysis is used to evaluate the correlation between the expression of 54,000 genes in 10,000 cancer samples and the survival rates of 21 different cancers. The samples include 371 livers, 1,440 stomachs, 2,190 ovaries, 3,452 lungs, 6,234 breast cancer and 543 UCEC samples. Use the Kaplan-Meier diagram^3^ to analyze the relationship between gene expression and survival rates of endometrial cancer through hazard ratio (HR), and Logarithmically sort the P value (Lánczky et al., 2016).

### GeneMANIA Database

GeneMANIA^4^ is mainly used to construct a protein-protein interaction (PPI) network, generate hypotheses about gene function, and determine the priority of genes by analyzing the gene list (Warde-Farley et al., 2010). Entering the target gene in the site to generate protein—protein interaction network, each small circles represent different proteins in the network, the size of the circle represents the strength of the interaction, different colors of the attachment has the validation of different means of interaction, the validation includes a variety of bioinformatics methods: physical interaction, gene co-expression, gene co-localization, gene enrichment analysis, and website prediction. Besides, the annotation information of the protein can also be queried in the target network.

### LinkedOmics Database

The LinkedOmics database^5^ is mainly used for comprehensive data analysis related to TCGA cancer 32 sets (Vasaikar et al., 2018). It also includes mass spectro-based proteomic data generated by the Clinical Proteomics Oncology Analysis Association (CPTAC) for TCGA breast, colorectal, and ovarian tumors. The LinkFinder module of LinkedOmics was used to study the differentially expressed genes related to RNF183 in the TCGA UCEC cohort (*n* = 176). The results provided by the database are shown in the form of a volcano map, heat map or scatter plot by Pearson correlation coefficient analysis. Besides, biological processes, cellular components, molecular functions, and enrichment, and analysis of KEGG pathways were performed through genomic enrichment analysis (GSEA). The grade standard is FDR < 0.05, and 500 simulations have been performed.

### TIMER Database

The TIMER database runs more than 10,000 samples from the Cancer Genome Atlas (TCGA) to systematically analyze the tumor infiltrating immune cells (TIIC) of 32 kinds of cancers^6^ (Li et al., 2017). TIMER determines the abundance of tumors by statistical analysis of gene expression profile, 106 infiltrated immune cells (TIIC) were analyzed (Li et al., 2016). The gene module is mainly used to explore the correlation between gene expression and immunoglobulin content. The survival module is applied to seek the relationship between clinical outcomes and immune infiltration or gene expression richness. Correlation between the mutated gene and the content of immune infiltration fluid from the mutation module. SCNA model is adopted to explore the correlation between somatic CNA and immune infiltration richness. The Diff Exp module is selected to examine the differential gene expression between tumor and normal tissues. The correlation module is used to research the correlation between genes. The Go Estimatio module can run private samples of users with the TIMER algorithm. We analyzed the relationship between RNF183 gene expression level and infiltrating immune cells by Spearman analysis (including B cells, CD4 + T cells, CD8 + T cells, neutrophils, dendritic cells, and macrophages).

### Plasmids and Antibodies

Anti-RNF183 antibody (1:1,000, NBP1-74192, Novus Biologicals, Colorado, United States), anti-ERα antibody (1:1,000, ab267512, Abcam, Cambridge, United Kingdom), anti-GAPDH antibody (1:3,000, 10494-1-AP, Proteintech Group, Chicago, United States), HRP-conjugated Affinipure Goat Anti-Mouse IgG (H + L) (1:10,000, SA00001-1 Proteintech Group, Chicago), HRP-conjugated Affinipure Goat Anti-Rabbit IgG (H + L) (1:10,000, SA00001-2 Proteintech Group) were used for western blot. RNF183 (pcDNA4-myc/his-RNF183) and RNF183 without amino acids 1–60 were illustrated previously (Geng et al., 2017). The ERE-TK-Luc and the pRL-TK plasmids were constructed by the Genewiz Company (Suzhou, China).

### Cell Culture

Ishikawa cells were cultured in RPMI-1640 (Gibco, Carlsbad, CA, United States) with 10% fetal bovine serum (FBS) (Gibco) plus 100 U/ml penicillin G, and 100μg/ml streptomycin (Gibco) in a humidified atmosphere of 5% CO2 at 37°C. Ishikawa cells were treated with 100μg/mL cycloheximide after transfected with siNC or siERα for 48 h. Ishikawa cells were transfected with siNC or siRNF183 followed by administrating 100 nM MG132 6 h.

### siRNA Transfection

The package of si-h-RNF183 and si-h-ESR1 were designed by RIBOBIO company (siRNA for RNF183 ID: SIGS0015614-1, siRNA for ESR1 ID: SIGS0005356-1, Beijing, China). 50% fusion Cells were transfected with 75 nM siRNAs using 5 μL Lipofectamine 2000 (Invitrogen, Grand Island, NY) in per six well-cell plates. The samples were collected after transfected 48 h.

### Quantitative PCR

RNAs were extracted using Trizol (Invitrogen). The cDNA was reversed from 1 μg RNA using M-MLV reverse transcriptase (Promega, Madison, WI, United States). qPCR was examined using SYBR Green (BIO-RAD, Hercules, CA, United States) for 40 cycles (95°C for 15 s, 60°C for 30 s). The primer sequence of mRNA for qPCR are available in Supplementary Table S1 and synthesized by Sangon Biotech (Shanghai, China).

### Luciferase Reporter Assay

Luciferase activity was assessed by the Dual-Luciferase Reporter Assay (Promega). Briefly, Ishikawa cells were transfected with siRNF183 or siNC or with pcDNA4-myc/his-RNF183 or RNF183△t or pcDNA4-myc/his vector along with ERE reporter plasmids. Cells were treated with E2 (10 nM) after 24 h post-transfection. After another 24 h, samples were collected for Luciferase activity measure.

### Western Blotting

Cells were lysed with RIPA lysis buffer (G-Clone) containing protease inhibitor (G-Clone). The concentration of protein was examined by BCA Protein Assay Kit (KeyGen BioTECH, Jiangsu, China). Collected lysates were resolved in 12% SDS-polyacrylamide gel and the protein was detected with the indicated antibodies.

### Statistical Analysis

Data were revealed as mean ± standard deviation (SD). The survival curve was generated by Kaplan-Meier plots relation analysis. The expression of related genes were evaluated using Pearson correlations. Other data were assessed using Student’s *t*-test. *P* < 0.05 was considered statistically significant.

## Results

### Clinical Relevance of RNF183 Expression in Endometrial Cancer

**FIGURE 1:**
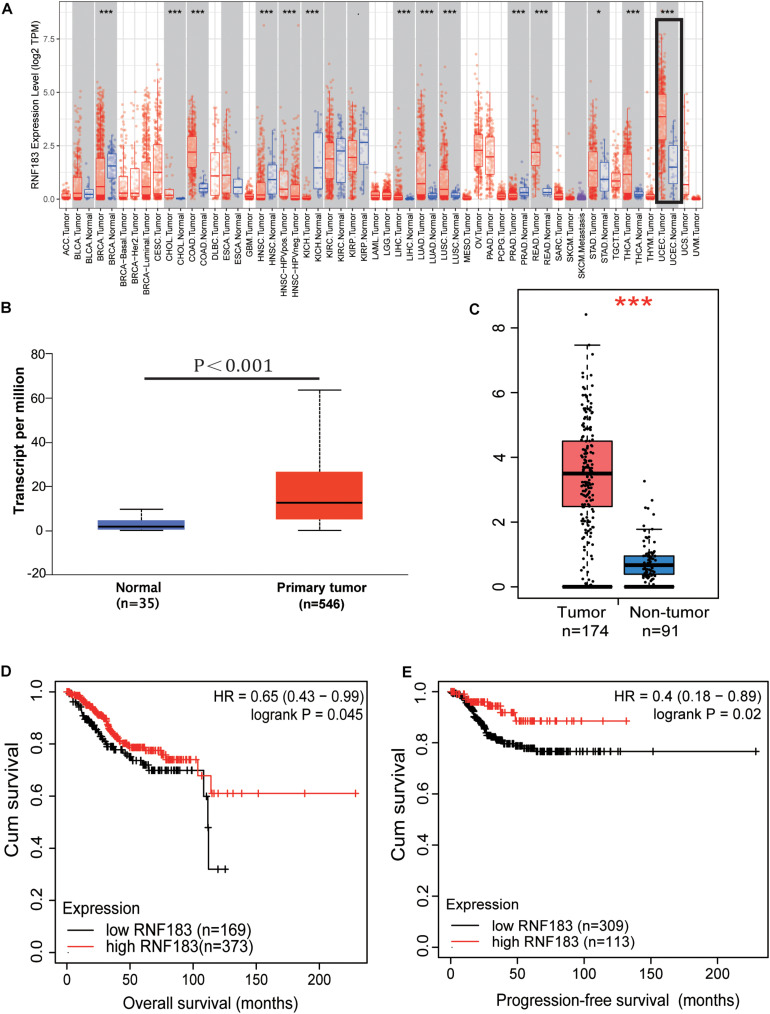
Expression of RNF183 in human endometrial cancer. **(A)** The differential expressions of RNF183 between normal and tumor tissues exist in the majority of human cancers (TIMER). **(B,C)** RNF183 was elevated in endometrial cancer tissues compared to normal endometrium (UALCAN and GEPIA). Increased expression of RNF183 is associated with favorable prognosis of overall survival **(D)** and progression-free survival **(E)** in TCGA patients stratified at “best cut-off” (Kaplan-Meier Plotter Database). *P<0.05, ***P<0.001.

From the TIMER database of the Diff Exp module across all the cancer genome atlas (TCGA) tumors, our studying showed that a high proportion of RNF183 exists in the majority of human cancer tissues (Figure 1A). Among all the cancer types, RNF183 is remarkably upregulated in endometrial cancer compared with normal endometrium. To investigate the role of RNF183 in endometrial cancer, we utilized UALCAN website to assess RNA-seq in 546 primary endometrial tumors and 35 normal endometrial tissues. RNF183 was shown to be elevated in cancerous tissues compared to normal endometrium (Figure 1B), which was accordant with statistics documented in GEPIA (Figure 1C).

Next, we were encouraged to apply the Kaplan-Meier Plotter online tool to explore the clinical importance of RNF183 in extensive RNA-seq data classifying patients based on the “best cut-off” value. RNF183 high expression was associated with favorable overall survival (OS, Figure 1D) and progression-free survival (PFS, Figure 1E) in 542 patients.

**FIGURE 2:**
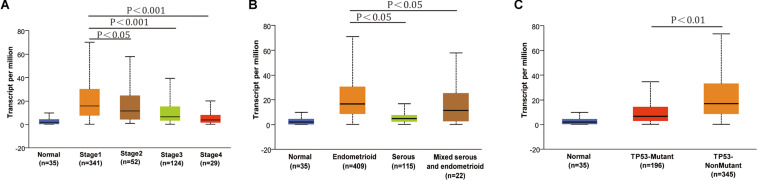
The expression of RNF183 in particular subtypes of endometrial cancer (UALCAN). **(A)** RNF183 mRNA level in normal individuals or endometrial tumor stage 1,2,3, or 4. **(B)** RNF183 mRNA expression in normal individuals or histological subtypes (Endometrioid, Serous, or Mixed serous and endometrioid). **(C)** RNF183 mRNA level in normal individuals, in TP53-Mutant or TP53-Non-Mutant tumors.

The conclusion above made us search RNF183 expression in different subtypes and tumor grades of endometrial cancer, which results in diversification of the disease and specific clinical outcomes. The results from UALCAN showed that RNF183 expression was significantly increased at stage 1 in comparison with other high-grade stages (Figure 2A). Additionally, we found that RNF183 level was considerably higher in endometrioid adenocarcinomas compared to non-endometrioid adenocarcinomas (Figure 2B). Meanwhile, TP53-Non-Mutant patients harbored high RNF183 expression compared to TP53-mutated patients (Figure 2C).

### RNF183 Co-expression Networks in UCEC

**FIGURE 3:**
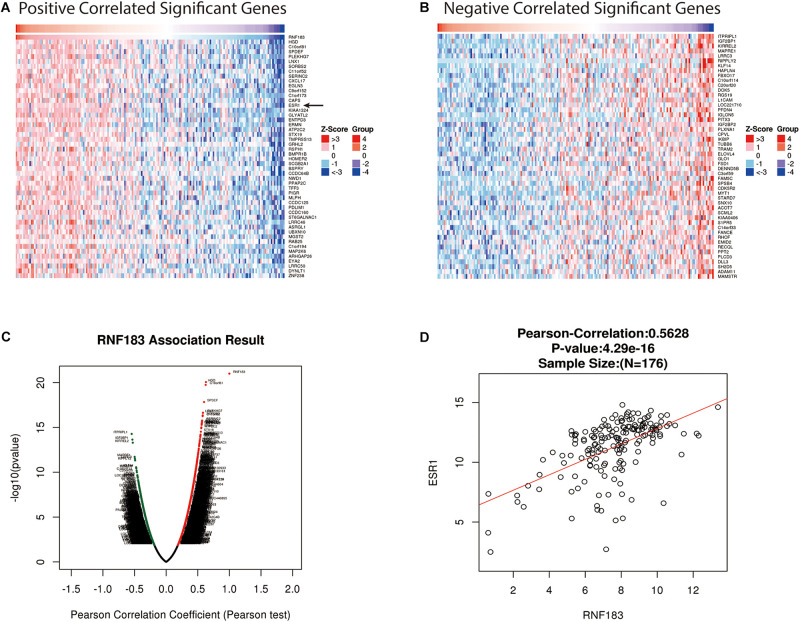
Genes differentially expressed in correlation with RNF183 in UCEC (LinkedOmics). **(A–B)** Heat maps showing genes positively and negatively correlated with RNF183 in UCEC (TOP 50). **(C)** A Pearson test was used to assess correlations between RNF183 and genes differentially expressed in UCEC. Red indicates positively correlated genes and green indicates negatively correlated genes. **(D)** The scatter plot shows the Pearson correlation of RNF183 expression with ESR1.

**FIGURE 4:**
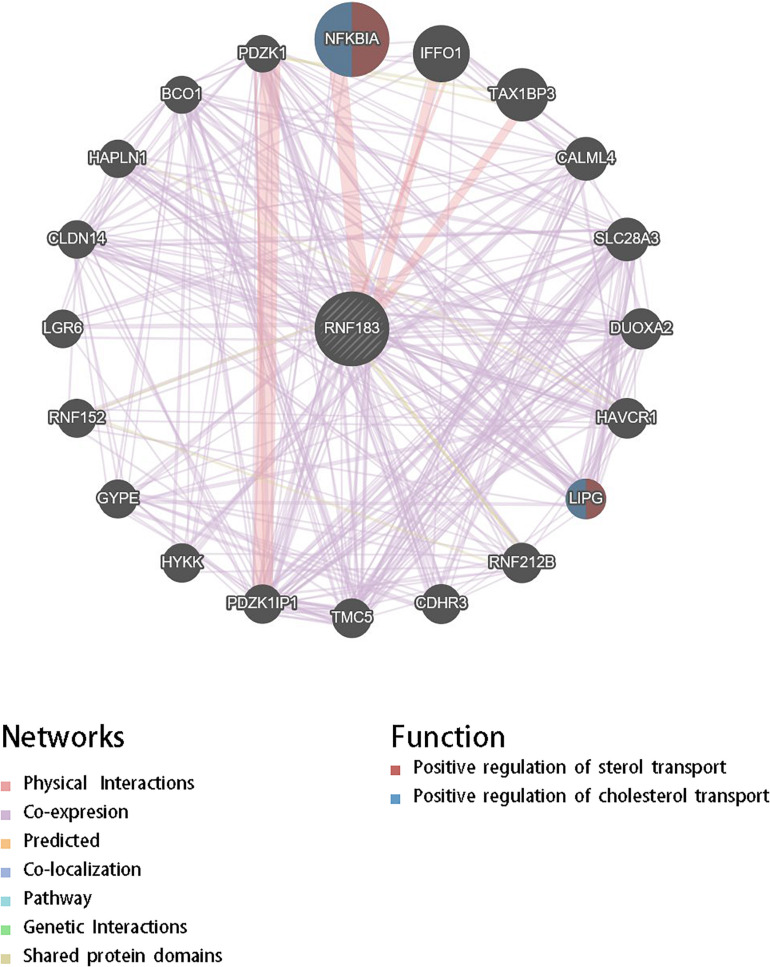
Protein-protein interaction network of RNF183 networks (GeneMANIA). Protein-protein interaction (PPI) network and functional analysis revealed the enrichment of the target gene set of RNF183. The different colors at the edges of the network represent the applied enrichment methods: physical interactions, co-expression, predicted, co-localization, pathways, genetic interactions, and shared protein domains. The different colors of the network nodes represent the biological functions of the enriched gene set.

For gaining insight into RNF183 biological meaning in UCEC, We used the function module of LinkedOmics to examine RNF183 co-expression mode in the UCEC cohort. As shown in Figure 3C, 8,777 genes (dark red dots) were demonstrated significant positive correlations with RNF183, whereas 11,121 genes (dark green dots) were shown significant negative associations (false discovery rate, FDR < 0.01). The top 50 significant genes positively and negatively correlated with RNF183 were shown in the heat map (Figures 3A,B). The statistical scatter plots for individual genes are shown in Figure 3D. Besides, we discussed the protein-protein interaction (PPI) network and the function of RNF183 through GeneMANIA (Figure 4).

### Enrichment Analysis of RNF183 Functional Networks in UCEC

**FIGURE 5:**
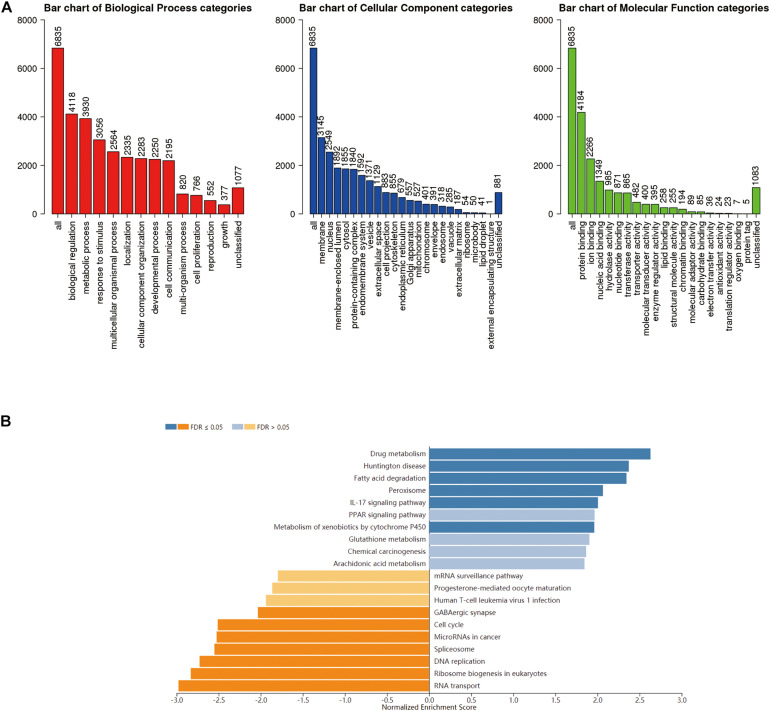
Enriched GO annotations and KEGG pathways of RNF183 correlated genes in UCEC (LinkedOmics). **(A)** Biological process, Cellular Component and Molecular function analysis. **(B)** KEGG pathway analysis. Dark blue and orange indicate FDR ≤ 0.05, light blue and orange indicate FDR > 0.05 in **(B)**. FDR, false discovery rate.

GO term analysis by gene set enrichment analysis (GSEA) showed that genes differentially expressed in correlation with RNF183 were located mainly in the membrane and nucleus, where they participate in biological regulation, metabolic process, and response to the stimulus. They act as protein binding, ion binding, and nucleic acid binding (Figure 5A). KEGG pathway analysis showed enrichment in the drug metabolism, Huntington disease, fatty acid degradation, peroxisome, IL-17 signaling pathway, and PPAR signaling pathway (Figure 5B).

### RNF183 Correlates With Tumor Purity and Immune Infiltration Level in UCEC

**FIGURE 6:**
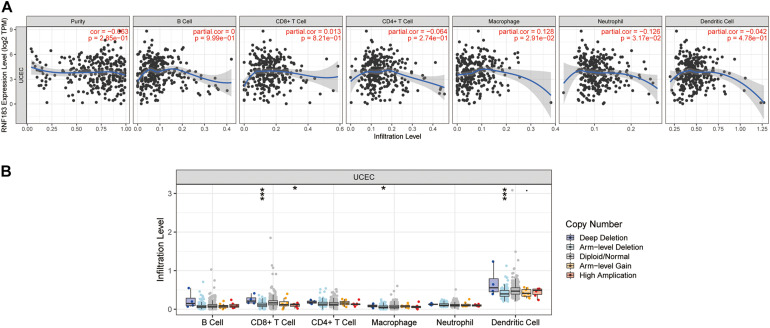
Correlations of RNF183 expression with immune infiltration level in UCEC (TIMER). **(A)** RNF183 expression is negatively related to tumor purity, infiltrating levels of CD4 + T cells, neutrophils, and dendritic cells and has positively correlations with infiltrating levels of macrophages in UCEC. **(B)** RNF183 CNV affects the infiltrating levels of CD8 + T cells, macrophages, and dendritic cells in UCEC. *P < 0.05, ***P < 0.001.

We investigated whether RNF183 expression was correlated with immune infiltration levels in UCEC from TIMER database. The results show that RNF183 expression has negatively correlations with tumor purity (*r* = −0.063, *p* = 2.85E–01), infiltrating levels of CD4 + T cells (*r* = −0.064, *p* = 2.74E–01), neutrophils (*r* = −0.126, *p* = 3.17E–02), and dendritic cells (*r* = −0.042, *p* = 4.78E–01) (Figure 6A). In addition, RNF183 CNV has significant correlations with infiltrating levels of CD8 + T cells, macrophages, and dendritic cells (Figure 6B).

### Correlation Analysis Between mRNA Levels of RNF183 and Markers of Different Subsets of Immune Cells

We further evaluated the relationship between the RNF183 level and immune infiltrating cells through the TIMER database based on the expression level of immune marker genes in UCEC tissues. The immune cells analyzed include CD8^+^ T cells, CD4^+^ T cells, B cells, monocytes, tumor-associated macrophages (TAM), M1 and M2 macrophages, neutrophils, and natural killer (NK) cells, dendritic cells, and besides, different subgroups of T cells, namely T helper 1 (Th1), Th2, Th17, regulatory T (Tregs), and T cell exhaustion. Because tumor purity will affect the level of immune infiltration of clinical samples, the purity of the relevant analysis was adjusted (Table 1).

**TABLE 1:** Correlation analysis between RNF183 and relate genes and markers of immune cells in TIMER.

**Description**	**Gene markers**	**UCEC**			
		**None**		**Purity**	
		**Cor**	**P**	**Cor**	**P**
CD8 + T cell	CD8A	–0.052	2.22e–01	–0.119	4.16e–02
	CD8B	–0.027	5.22e–01	–0.031	6.02e–01
T cell (general)	CD3D	–0.046	2.82e–01	0	9.98e–01
	CD3E	–0.037	3.86e–01	–0.027	6.49e–01
	CD2	–0.015	7.22e–01	–0.018	7.64e–01
B cell	CD19	–0.026	5.52 e–01	–0.051	3.81e–01
	CD79A	–0.023	5.88e–01	–0.121	*
Monocyte	CD86	–0.094	*	–0.087	1.36e–01
	CD115	–0.025	5.57e–01	–0.011	8.54e–01
TAM	CCL2	–0.013	7.85e–01	0.048	4.09e–01
	CD68	–0.07	1.01e–01	–0.066	2.60e–01
M1 Macrophage	iNOS	–0.26	7.61e–01	–0.256	***
	IRF5	–0.074	8.38e–02	–0.107	6.65e–02
	COX2	–0.12	**	0.085	1.46e–01
M2 Macrophage	CD163	–0.183	***	–0.182	**
	VSIG4	–0.114	**	–0.122	*
	MS4A4A	–0.131	**	–0.146	*
Neutrophils	CD66b	0.07	1e–01	0.023	6.92e–01
	CD11b	0.089	*	0.092	1.16e–01
	CCR7	0.014	7.44e–01	–0.047	4.18e–01
Natural killer cell	KIR2DL1	0.028	5.16e–01	0.005	9.34e–01
	KIR2DL3	0.028	5.17e–01	–0.029	6.27e–01
	KIR2DL4	0.039	3.67e–01	–0.032	5.82e–01
	KIR3DL1	0.045	2.94e–01	0.016	7.81e–01
	KIR3DL2	–0.74	8.43e–02	–0.027	6.41e–01
	KIR3DL3	–0.004	9.23e–01	–0.034	5.62e–01
	KIR2DL4	0.039	3.67e–01	–0.032	5.82e–01
Dendritic cell	HLA-DPB1	0.083	5.27e–02	0.055	3.44e–01
	HLA-DQB1	0.134	**	0.099	9.00e–02
	HLA-DRA	0.144	***	0.125	*
	HLA-DPA1	0.051	2.33e–01	0.018	7.60e–01
	BDCA-1	0.275	***	0.249	***
	BDCA-4	0.219	***	0.172	**
	CD11c	0.094	*	0.098	9.52e–02
Th1	T-bet	0.025	5.53e–01	–0.048	4.16e–01
	STAT4	0.067	1.18e–01	–0.037	5.29e–01
	STAT1	–0.256	***	–0.252	***
	IFN-γ	–0.104	*	–0.138	**
	TNF-α	0.044	3.06e–01	0.004	9.50e–01
Th2	GATA3	–0.113	**	–0.217	***
	STAT6	0.25	***	0.197	***
	STAT5A	0.014	7.47e–01	–0.009	8.73e–01
	IL13	–0.021	6.18e–01	0.007	9.90e–01
	IL21	–0.007	8.69e–01	–0.058	3.23e–01
Th17	STAT3	0.202	***	–0.156	7.43e–03
	IL17A	0.005	9.15e–01	0	9.96e–01
Treg	FOXP3	0.008	8.6e–01	–0.083	1.57e–01
	CCR8	0.067	1.18e–01	–0.001	9.93e–01
	STAT5B	0.035	4.17e–01	–0.076	1.97e–01
	TGFβ	–0.172	***	–0.251	***
T cell exhaustion	PD-1	–0.12	**	–0.144	**
	CTLA4	0.038	3.72e–01	–0.002	9.78e–01
	LAG3	–0.222	***	–0.296	***
	TIM-3	–0.078	6.81e–02	–0.097	9.62e–02
	GZMB	–0.151	***	–0.172	**

TAM, tumor-associated macrophage; Th, T helper cell; Treg, regulatory T cell; Cor, P value of Spearman’s correlation; None, correlation without adjustment. Purity, correlation adjusted by purity. *P < 0.05, **P < 0.01, ***P < 0.001.

Specifically, RNF183 expression showed significant correlation with the expression of markers of specific immune cells such as B cell, CD79A (*r* = −0.121; *P* = 3.84e–02), M1 macrophage marker, iNOS (*r* = −0.256; *P* = 9.01e–06), M2 Macrophage, CD163 (*r* = −0.182; *P* = 1.77e–03), VSIG4 (*r* = −0.122; *P* = 3.71e–02), MS4A4A (*r* = −0.146; *P* = 1.21e–02), Dendritic cell, HLA-DRA (*r* = −0.182; *P* = 3.30e–02), BDCA-1 (*r* = −0.249; *P* = 1.60e–05), BDCA-4 (*r* = −0.172; *P* = 3.15e–03). The expression of RNF183 correlated significantly with the expression of the marker genes of different subsets of T cells in UCEC, namely, Th1 markers, STAT1 (*r* = −0.252; *P* = 1.25e–05), IFN-γ (*r* = −0.138; *P* = 1.80e–02), Th2 markers, GATA3 (*r* = −0.217; *P* = 1.77e–04), STAT6 (*r* = −0.197; *P* = 6.96e–04), Treg markers, TGFβ (*r* = −0.251; *P* = 1.37e–05), T cell exhaustion markers, PD-1 (*r* = −0.217; *P* = 1.34e–02), LAG3 (*r* = −0.223; *P* = 5.83e–02), GZMB (*r* = −0.172; *P* = 3.31e–03). RNF183 expression did not show any significant correlation with the expression of marker genes for CD8^+^ T cells, T cell (general), Monocyte, TAM, Neutrophils, Natural killer cell and Th17 cells. These results demonstrated RNF183 expression were associated with infiltration of immune cells in UCEC (Table 1).

### RNF183 Modulates ERα Expression of ERα Positive Endometrial Cancer Cell

**FIGURE 7:**
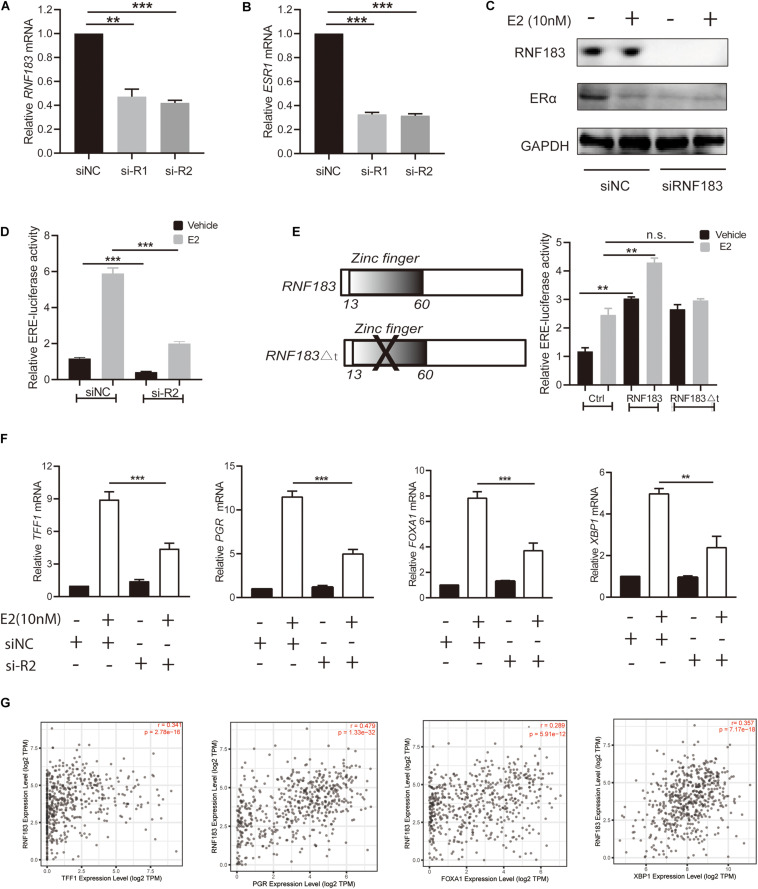
RNF183 controls ERα expression in endometrial cancer. **(A)** Ishikawa cells transfected with siRNF183 or siNC and RNF183 knockdown efficiency were examined by RT-PCR. **(B)** ESR1 mRNA level decreased in the Ishikawa cell line after transfection with siRNF183. **(C)** The protein level of ERα was downregulated based on RNF183 deletion. **(D)** RNF183 deletion decreased ERα-dependent expression of the ERE-luciferase activity. **(E)** The ERE-Luciferase activity was evaluated in Ishikawa cells with overexpression of pcDNA4-myc/his-RNF183 or pcDNA4-myc/his vector or truncated RNF183 without E3 ubiquitin ligase activity (13–60 amino acids). **(F)** Diminished E2 induced reduction of ERα target genes following inhibition of RNF183 with siRNA. **(G)** RNF183 positively associated with TFF1, PGR, FOXA1 and XBP1 from the TIMER database. Experiments were repeated in triplicates. Mean ± S.D. (*n* = 3). **P<0.01, ***P<0.001.

Bioinformatics analysis via LinkedOmics, We found RNF183 was markedly positively correlated with ESR1 (Figure 3D). To verify this finding, we used the ERα-positive Ishikawa cell line as a model. Upon silencing of RNF183 using two different individual small interfering RNA, we detected a noticeable reduction in ESR1 mRNA levels (Figure 7B), and the knockdown efficiency was shown in Figure 7A. Under stimulating E2 (17β-estradiol) or vehicle (absolute ethanol) conditions, ERα protein levels were also diminished following RNF183 silencing (Figure 7C). To determine the mechanism through which RNF183 regulates ERα, we assayed ERα luciferase reporter activity following RNF183 depletion or RNF183 overexpression. Figure 7D shows that the RNF183 knockdown suppressed the activity of the ERα reporter gene. While overexpression of RNF183 resulted in the raised activity of the ERα reporter gene no matter existence or absence of E2 stimulation (Figure 7E). As ubiquitin ligase has been reported to engage in the process of transcription upon the structure of the zinc finger domain (Molloy et al., 2018), we generated a curtailed form of RNF183 (RNF183△t) without E3 ubiquitin ligase activity by deleting zinc finger domain (amino acids 1–60). This truncation mostly canceled the function of RNF183 in stimulating the activity of the ERα luciferase report gene (Figure 7E). RNF183 depletion also reduced the expression of endogenous ERα target genes dependent on E2 stimulation such as TFF1, PGR, FOXA1, and XBP1 (Figure 7F). Furthermore, TFF1, PGR, FOXA1, and XBP1 showed markedly positive correlation with RNF183 from TIMER database (Figure 7G).

### ERα Mediates RNF183 Stability in ERα Positive Endometrial Cancer Cell

**FIGURE 8:**
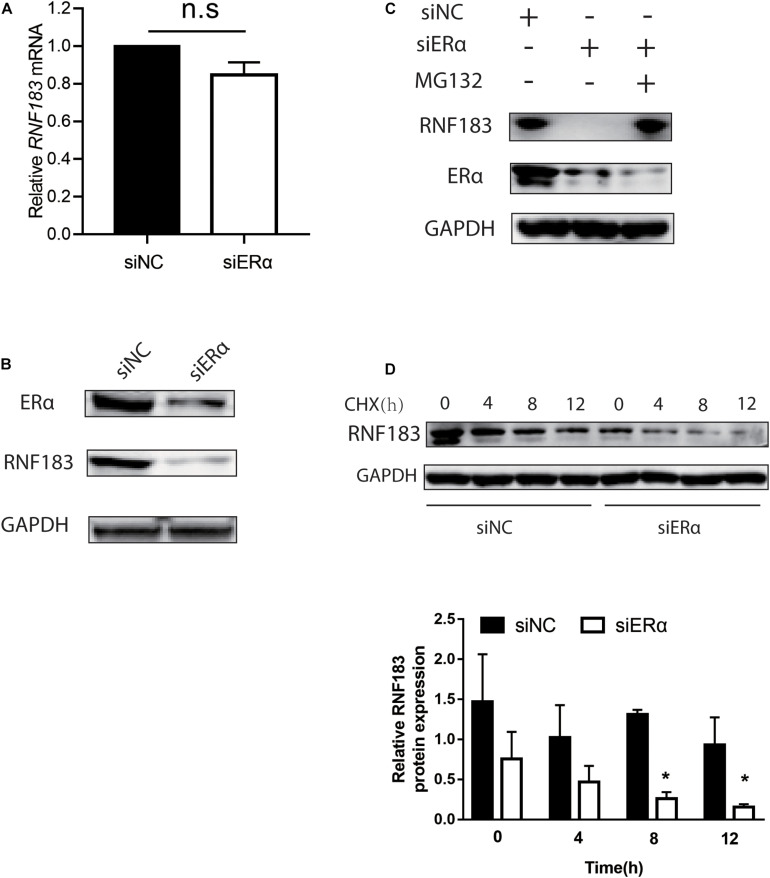
ERα regulates RNF183 expression and increases its stability. The expression of endogenous RNF183 mRNA **(A)** and protein levels **(B)** in Ishikawa cells after transfecting with siERα or siNC. **(C)** Downregulated RNF183 protein level reduced by siERα was recovered based on MG132 treatment. **(D)** Depletion ERα weakens RNF183 stability. RNF183 protein level examined at indicated time after transfected with siERα 48h followed 100μg/mL cycloheximide treatment. Experiments were repeated in triplicates. Mean ± S.D. (*n* = 3). *P<0.05.

Given that ERα has been shown to participate in the feedback loop with some enzymes and transcription factors (Eeckhoute et al., 2007; Molloy et al., 2018), the impact of ERα on the expression of RNF183 was analyzed in the Ishikawa cell line. We noticed that ERα knockdown had little effect on the mRNA level of RNF183 (Figure 8A). However, there was a marked decline in the RNF183 protein level (Figure 8B). Next, in the presence of proteasome inhibitor MG132, RNF183 was in a stable state, even being with siERα (Figure 8C). Furthermore, ERα inhibition expedited the reduction of RNF183 protein expression in the presence of protein synthesis inhibitor cycloheximide (Figure 8D). In sum, these data indicate that ERα raises RNF183 protein stability in the ERα-positive endometrial cancer cells.

## Discussion

RNF183 has been reported to occur in diverse diseases such as colorectal cancer (CRC), kidney disease, inflammatory bowel disease and various biological processes. RNF183 stimulated inflammatory bowel disease progression (Yu et al., 2016). RNF183 was also identified as an oncogene promoting proliferation, metastasis, and a resistance gene for trametinib in CRC cells via activating the NF-κB signal (Geng et al., 2017). In the renal medullary collecting duct, specific RNF183 controlled cell adaption to hypertonic stress by regulating Na, K-ATPase level (Maeoka et al., 2019b; Okamoto et al., 2020b). Under physiological condition, RNF183 localizing on the endoplasmic reticulum, interacted and ubiquitin-mediated degradation of Bcl-xL, suggesting a crucial role of RNF183 in executing programmed cell death (Wu et al., 2018). The results of our study showed that significantly amplification of RNF183 was considered as a prognostic marker in endometrial cancer. Analysis implied that among endometrial cancer, High RNF183 expression seems to associate with low stage, endometrioid and TP53-Non-Mutant status, which are usually with a good prognosis. Also, the RNF183 expression was greater at higher expression and the tumor stage was greater at the lower level, implying the early role of RNF183 in the development of endometrial cancer.

Based on the marker levels of different immune cell types in UCEC, RNF183 mRNA level is correlated with the number of tumor infiltrating immune cells, which indicates that RNF183 plays a vital role in regulating tumor immunity. We observed that the expression level of RNF183 mRNA was negatively correlated with CD4 + T cells, neutrophils, and dendritic cells. We also observed the correlation between the levels of RNF183 mRNA and the expression of the B cell (CD79A), M1 macrophage marker (iNOS), M2 Macrophage (CD163, VSIG4, and MS4A4A), Dendritic cell (HLA-DRA, BDCA- 1, and BDCA-4). The expression of RNF183 is also related to the markers in different subgroups of T helper (Th) cells, including Th1 (STAT-1, and IFN-γ), Th2 (GATA3 and STAT6), Treg (TGF-β), T cell exhaustion markers (PD-1, LAG3, and GZMB). Above indicate the role of RNF183 in regulating tumor invasion of T helper cells.

Moreover, the depleted T cell markers PD-1, LAG3 and GZMB, which are critical inhibitory immune checkpoint proteins, are negatively correlated with the expression of RNF183. The expression of PD-1 (Kucukgoz Gulec et al., 2019) is considered a sign of poor prognosis of endometrial cancer and it has been widely studied as a target of immunotherapy. LAG3 (Friedman et al., 2020) can be used as a target for immunotherapy in endometrial cancer and in conjunction with other immune checkpoints, such as PD-1. Besides, Granzyme B + cells (Pakish et al., 2017) have increased expression in high microsatellite instability (MSI-H) endometrial cancer, providing a therapeutic target for immunotherapy. We speculate that RNF183, which is highly expressed in the tumor microenvironment, leads to a better prognosis of UCEC by regulating the expression of inhibitory immune checkpoint proteins PD-1, LAG3 and GZMB on exhausted T cells. However, this assumption needs further verification.

Through heatmap about the top 50 genes positively correlated with RNF183, we found ESR1 was one of the most notable positive genes with RNF183. Most endometrial cancers are estrogen-related endometrioid adenocarcinomas. Beyond 90% endometrioid carcinoma express moderate to high levels of the ERα (gene symbol ESR1) (Lebeau et al., 2008; Smith et al., 2018). A consensus that patients with tumor positive ERα expression have a favorable prognosis of endometrial cancer (Creasman et al., 1980; Iversen et al., 1988; Jongen et al., 2009; Supplementary Figure S1). To confirm the regulatory relationships between RNF183 and ERα, We used ERα-positive cell line Ishikawa as a model to examine. We clarify a character for RNF183 in promoting ERα expression at the transcript and protein level in endometrial cancer. ERα is a substrate for E3 ubiquitin ligase (Byun and Jung, 2008; Zhang et al., 2015). We proved that RNF183 controls ERα activity determined by the RING finger domain. The transcriptional activation of estrogen bound ERα is tissue-specific (Kushner et al., 2000; Castro-Rivera and Safe, 2003). PGR (Progesterone receptor), FOXA1 (Forkhead box protein A1), XBP1 (X-box binding protein 1), and TFF1 (Trefoil factor 1) were reported to involve in the estrogen signal in endometrial cancer (Baxter et al., 2019). Clinical samples favor RNF183 positively correlated with TFF1, FOXA1, XBP1, and PGR.

Noticeable studies showed that ERα often participates in the positive regulation with the related gene, such as autocrine loop of the CXCR4/SDF-1 and ERα/ERβ signaling pathways (Sauvé et al., 2009), S6K1-ERα and ER-α36/EGFR positive feed-forward loop (Zhang et al., 2011; Maruani et al., 2012). We found that although the essential role of ERα is a transcription factor, knockdown ERα does not affect the mRNA level of RNF183, but a decreased in protein level. ERα could mediate ubiquitination and protein degradation had been reported (Lai et al., 2019). Our further results demonstrated that RNF183 protein level recovery followed proteasome inhibitor MG132 treated signifying ERα impeded RNF183 reduction by the inhibition of the proteasome, and ERα depletion stimulated RNF183 degradation.

## Conclusion

Our results indicate that RNF183 is a potential independent prognostic biomarker of UCEC, which can also be used to assess the level of immune cell infiltration in tumor tissues. Furthermore, ERα plays a vital role in the histology and progression of endometrial cancer. We found that RNF183 seems to be a new marker associated with ERα in ERα-positive endometrial cancer. Furthermore, the crosstalk between RNF183 and ERα may be the reason for the abnormally high expression of RNF183 in endometrial cancer.

## Data Availability Statement

The original contributions presented in the study are included in the article/Supplementary Material, further inquiries can be directed to the corresponding authors.

## Author Contributions

LRM, GXL, and GR: conceptualization. LRM and GXL: resources. GR, ZLJ, HXB, QR, and ZRJ: formal analysis and investigation. GR and ZYH: data curation. GR: writing—original draft preparation, funding acquisition. ZYH: writing—review and editing, supervision. All authors have read and agreed to the published version of the manuscript.

## Conflict of Interest

The authors declare that the research was conducted in the absence of any commercial or financial relationships that could be construed as a potential conflict of interest.

## Footnotes

1 http://ualcan.path.uab.edu

2 http://gepia.cancer-pku.cn/index.html

3 http://kmplot.com/analysis/

4 http://www.genemania.org

5 http://www.linkedomics.org/login.php

6 https://cistrome.shinyapps.io/timer/

## Supplementary Material

Supplementary Figure 1 ERα is a strong predictor for prognosis in endometrial cancer. Patients with high expression of ERα harbor good OS **(A)** and PFS **(B)**.

Supplementary Table 1 The primer sequence of mRNA for qPCR in article.
