# Supplementary figures and images for: RNF183 Is a Prognostic Biomarker and Correlates With Tumor Purity, Immune Infiltrates in Uterine Corpus Endometrial Carcinoma
Source: Front Genet. 2020 Nov 26;11:595733. doi: 10.3389/fgene.2020.595733 (PMC7726321; doi:10.3389/fgene.2020.595733)

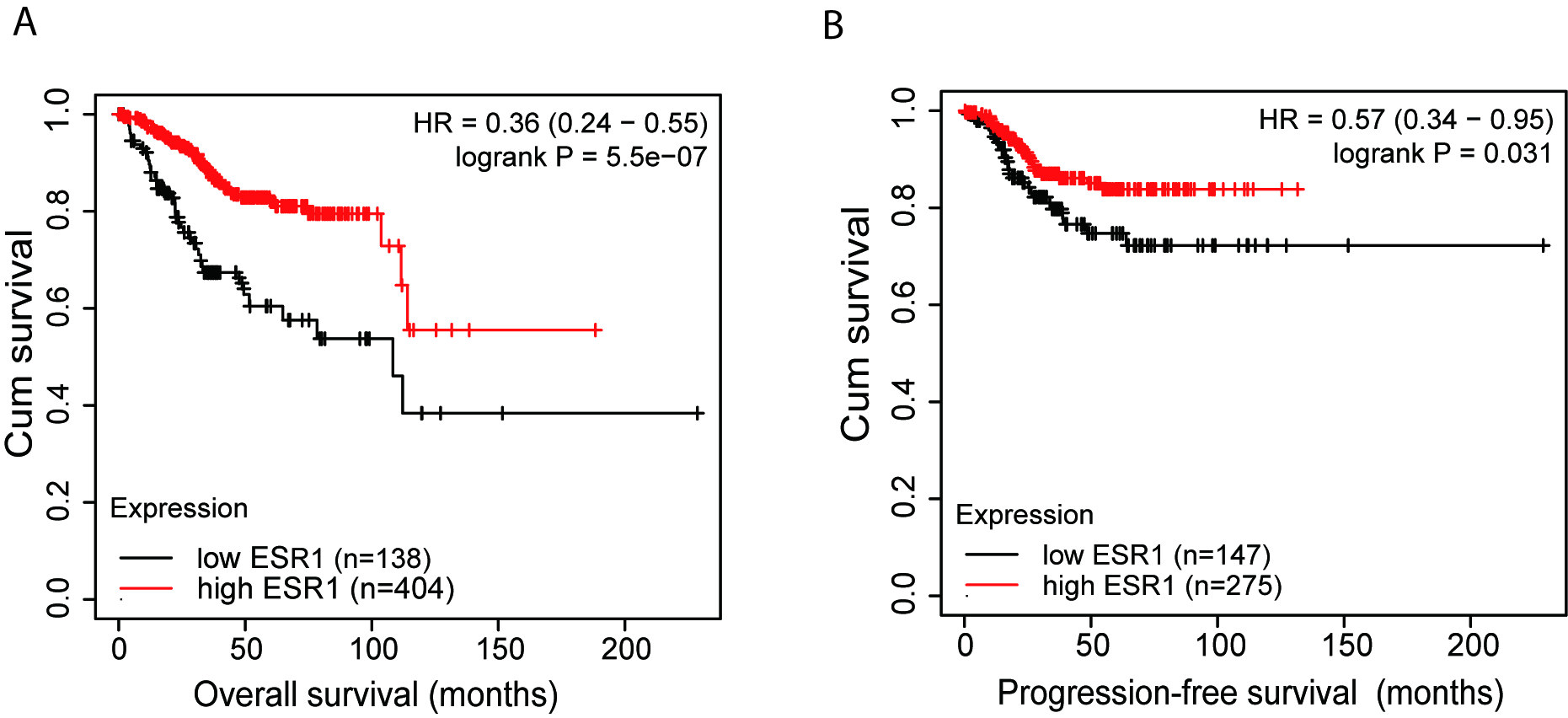

Supplement: Supplementary Figure 1 — ERα is a strong predictor for prognosis in endometrial cancer. Patients with high expression of ERα harbor good OS (A) and PFS (B). [file Image_1.TIF]
