# Supplementary material for: RNF183 Is a Prognostic Biomarker and Correlates With Tumor Purity, Immune Infiltrates in Uterine Corpus Endometrial Carcinoma
Source: Front Genet. 2020 Nov 26;11:595733. doi: 10.3389/fgene.2020.595733 (PMC7726321; doi:10.3389/fgene.2020.595733)
Supplement: Supplementary Table 1 — The primer sequence of mRNA for qPCR in article. [file Table_1.DOCX]

**Table S1** | The primer sequence of mRNA for qPCR in article

| **Genes** | **Sense primer** | **Antisense primer** |
| --- | --- | --- |
| RNF183 | 5’-CGAAAAGCTTGAAGGACTGG-3’ | 5’-TGAAGCAGCTCCAGTGAGAA-3’ |
| ESR1 | 5’- TCCTCATCCTCTCCCACATC -3’ | 5’- TCCAGCAGCAGGTCATAGAG-3’ |
| TFF1 | 5’- GCCACCATGGAGAACAAGGT -3’ | 5’- CAATTCTGTCTTTCACGGGG -3’ |
| PGR | 5’- CCAGGATTTCGGAATTT -3’ | 5’- GACACAGTGAATAGAACG -3’ |
| FOXA1 | 5'- GTGGCTCCAGGATGTTAGGA-3' | 5'- GAGTAGGCCTCCTGCGTGT-3' |
| XBP1 | 5'- ACACGCTTGGGAATGGACAC -3' | 5'- CCATGGGAAGATGTTCTGGG -3' |
| GAPDH | 5’-CTCCTCCTGTTCGACAGTCAGC-3’ | 5’-CCCAATACGACCAAATCCGTT-3’ |
